# Permissible Variation in the 3′ Non-Coding Region of the Haemagglutinin Genome Segment of the H5N1 Candidate Influenza Vaccine Cirus NIBRG-14

**DOI:** 10.1371/journal.pone.0036241

**Published:** 2012-05-11

**Authors:** Rachel E. Johnson, Michelle Hamill, Ruth Harvey, Carolyn Nicolson, James S. Robertson, Othmar G. Engelhardt

**Affiliations:** 1 Division of Virology, National Institute for Biological Standards and Control, Health Protection Agency, Blanche Lane, Potters Bar, Hertfordshire, United Kingdom; 2 BioStatistics, National Institute for Biological Standards and Control, Health Protection Agency, Blanche Lane, Potters Bar, Hertfordshire, United Kingdom; Johns Hopkins University - Bloomberg School of Public Health, United States of America

## Abstract

The candidate H5N1 vaccine virus NIBRG-14 was created in response to a call from the World Health Organisation in 2004 to prepare candidate vaccine viruses (CVVs) to combat the threat of an H5N1 pandemic. NIBRG-14 was created by reverse genetics and is composed of the neuraminidase (NA) and modified haemagglutinin (HA) genes from A/Vietnam/1194/2004 and the internal genes of PR8, a high growing laboratory adapted influenza A(H1N1) strain. Due to time constraints, the non-coding regions (NCRs) of A/Vietnam/1194/2004 HA were not determined prior to creating NIBRG-14. Consequently, the sequence of the primers used to clone the modified A/Vietnam/1194/2004 HA was based upon previous experience of cloning H5N1 viruses. We report here that the HA 3′ NCR sequence of NIBRG-14 is different to that of the parental wild type virus A/Vietnam/1194/2004; however this does not appear to impact on its growth or antigen yield. We introduced additional small changes into the 3′NCR of NIBRG-14; these had only minor effects on viral growth and antigen content. These findings may serve to assure the influenza vaccine community that generation of CVVs using best-guess NCR sequences, based on sequence alignments, are likely to produce robust viruses.

## Introduction

Influenza A viruses have a single-stranded negative sense RNA genome composed of eight individual RNA segments. Each segment is comprised of one or more open reading frames (ORFs) flanked by non-coding regions (NCRs) at their 3′ and 5′ ends. The 3′ and 5′ NCRs of influenza A viruses are important for virus replication [1]–[3] with the terminal 12 or 13 nucleotides of the 3′ and 5′ NCRs, respectively, being highly conserved among the eight RNA segments and different influenza A virus strains [1], [4]–[6]. Beyond these conserved nucleotides the sequences of the NCRs are segment specific with variable levels of conservation amongst different viruses. Sequencing the ends of RNA molecules is technically demanding; 5′ end sequences can be determined by direct sequencing of the RNA using reverse transcriptase whilst the 3′ ends require more sophisticated approaches such as the ‘RNA ligation’ method [7]–[9]. Thus, with the bulk of influenza virus sequencing focussing on the internal coding regions, the NCRs are largely ignored except by those researchers specifically interested in them. Also, when sequence information is available in the public domain for the NCRs, information regarding their derivation is often unavailable. It is therefore frequently not possible to regard the NCR sequences as genuine as it is not known whether the sequences have been determined *de novo* or represent the sequence of primers used during PCR and/or cloning.

The lack of influenza NCR sequences in the public domain and the lack of information regarding the provenance of published NCR sequences is a problem when they are required for primer design for specific genome segment amplification of novel viruses. Consequently, primers may often be designed based upon incorrect or incomplete information. This is a concern when novel candidate influenza vaccine viruses (CVVs) need to be developed rapidly, for example at the onset of an influenza pandemic.

The CVV NIBRG-14 was developed in 2004 at the National Institute for Biological Standards and Control (NIBSC) [10] following an urgent call from the World Health Organisation (WHO) to generate CVVs derived from highly pathogenic avian H5N1 viruses. NIBRG-14 was created by reverse genetics in the manner described by Subbarao et al., [11] and is composed of the NA and modified HA from A/Vietnam/1194/2004 and the internal genes of A/Puerto Rico/8/34 (PR8), a high growing laboratory adapted influenza A(H1N1) strain. Full length plasmid clones of A/Vietnam/1194/2004 HA and NA genome segments had to be generated with no prior knowledge of the precise sequence of the NCRs; instead primers used were based upon consensus sequences of H5N1 HAs and NAs available in public databases and which had been used successfully to generate at least one H5N1 virus [10]. In this study, the NCRs of the HA genome segment of wild type A/Vietnam/1194/2004 were determined and compared with those of NIBRG-14 and of other highly pathogenic H5N1 viruses from public databases. From these analyses viruses with modified NCRs were created, and the effects of these modifications on virus growth and HA content, important attributes of CVVs, were assessed.

**Figure 1:**
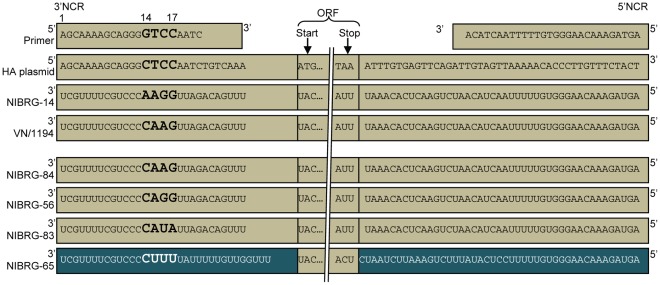
NCR sequences of primers, plasmids and viruses in this study . The primers used to clone A/Vietnam/1194/2004 haemagglutinin, the HA genome segment contained in the reverse genetics plasmid used to make NIBRG-14, RNA segment 4 (HA) of NIBRG-14 and of viruses generated or used in this study are shown schematically. Sequences are shown in positive sense for the plasmid and 3′ primer, and in negative sense for vRNAs. Terminal sequences of the 3′ and 5′ends of the HA vRNA for NIBRG-14, A/Vietnam/1194/2004 (VN/1194), NIBRG-84, NIBRG-56, NIBRG-83 and NIBRG-65 were determined from viral RNA by RNA ligation. Sequences on beige background indicate sequences derived from the HA segment of NIBRG-14, blue background represents sequences derived from PR8.

## Materials and Methods

### Modified HA Plasmids

A/Vietnam/1194/2004 HA genome segments with modifications in their 3′ NCR were generated by PCR using primers designed to introduce the desired sequence changes. All primers had SapI restriction sites at their 5′ ends. The amplified genome segments were inserted into pPST (a reverse genetics RNA transcription vector) using SapI restriction enzyme sites and standard cloning protocols [10]. All modified HA genes were based on the HA genome segment of NIBRG-14 which contains a deletion of the multibasic cleavage site.

**Figure 2:**
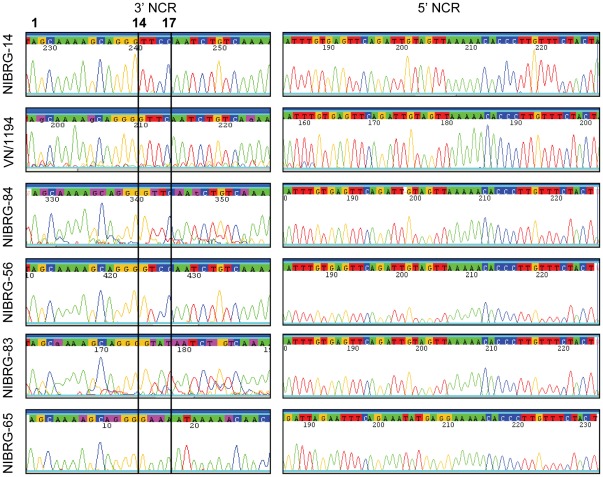
Sequencing of HA segment NCRs. Viral RNA was ligated and subjected to RT-PCR amplification as described in Materials and Methods and the sequence of the resulting RT-PCR products was determined by dideoxynucleotide sequencing. Sequencing traces covering the 3′ and 5′ vRNA NCRs are shown. Vertical lines indicate positions 14–17, counted from the 3′ end.

### Viruses and Cells

NIBRG-14 and NIBRG-65, derived by reverse genetics, have been described previously [10], [12]. The other H5N1 viruses used in this study were also generated by reverse genetics using modified HA segments containing point mutations in the 3′ vRNA NCR, the NA from A/Vietnam/1194/2004 and six genes from PR8. Viruses were characterised by sequencing of their HA and NA genes.

MDCK cells had been donated by the Common Cold Unit, Salisbury, in 1982 and were maintained in Eagle’s MEM +10% FBS [13].

### RNA Lligation

For determination of the sequence of the NCRs of the HA RNA genome segment, viral RNA was circularised by RNA ligation [7]–[8]. Viral RNA was extracted from 200 µl of infectious allantoic fluid in 1 ml Trizol containing 2 µl glycogen (50 mg/ml) and incubated at room temperature for 5 min. After centrifugation in a bench top centrifuge (13,000 rpm, 10 min, 4°C) the top layer was removed and added to 500 µl propanol and incubated at room temperature for 10 min. RNA was then pelleted in a bench top centrifuge (13,000 rpm, 15 min, 4°C), washed in 70% ethanol, air dried and resuspended in 20 µl water. The 5′ end of the RNA was dephosphorylated by adding 5 µl RNA to 0.5 µl of tobacco acid pyrophosphatase (TAP), 1 µl 10X TAP buffer, 2 µl Madin-Darby Canine Kidney (MDCK) cellular RNA and water to a total volume of 10 µl. This was incubated for 1 hr at 37°C and the enzyme was denatured for 5 min at 95°C. RNA was re-purified using Trizol as described above, and resuspended in 20 µl water. RNA ligation was performed with a 7 µl aliquot of de-phosphorylated RNA added to 1 µl T4 RNA ligase, 1 µl 10 mM ATP and 1 µl 10× T4 RNA ligase buffer, incubated for 1 hr at 37°C, and the enzyme was denatured for 5 min at 95°C. cDNA synthesis across the junction of the ligated HA RNA was performed using 4.7 µl ligated RNA, 0.5 µl Superscript II reverse transcriptase (100 U), 1.5 µl 100 mM DTT, 1.5 µl primer (3.2 pM), 3 µl 5× dNTP, 3 µl 5× Superscript II buffer and water to a total volume of 15 µl. The RT reaction was incubated for 1 hr at 42°C and enzyme denatured for 5 min at 95°C. PCR was then performed to amplify a DNA fragment spanning the junction of the ligated RNA and reactions were purified. The PCR products were sequenced to determine the sequence of the NCRs. The MDCK RNA used in the 5′ RNA dephosphorylation step was extracted from 75 cm^2^ flasks of MDCK cells that were trypsinised and pelleted at 2,000 rpm for 5 min. The pellet was then treated with Trizol and glycogen as described above using the same incubation and centrifugation steps and RNA was resuspended in 20 µl water per 75 cm^2^ flask. The sequence of the complete HA segment, including the non-coding regions, of wt A/Vietnam/1194/2004 has been deposited in the GISAID database (accession number EPI347615).

### Virus Concentrates

Viruses were grown in the allantoic cavity of 11-day-old embryonated hens’ eggs. Allantoic fluid was harvested 72 hours post infection and clarified by centrifugation (4,000 rpm, 30 minutes, 4°C, Beckman JS 5.3 rotor). A second clarification was performed (10,000 rpm, 30 minutes, 4°C, Beckman SW28 rotor) and virus was pelleted from clarified fluid by centrifugation (19,000 rpm, 90 minutes, 4°C, Beckman T19 rotor). Virus pellets were resuspended in 10 ml PBS and loaded over 10 ml 30% sucrose in SW28 tubes. Virus was pelleted by centrifugation (25,000 rpm for 90 min at 4°C, Beckman SW28 rotor) and resuspended in 100–200 µl PBS.

Work with 11-day-old embryonated hens’ eggs was carried out under a UK Home Office Project Licence and approved by the NIBSC Ethical Review Committee.

### SDS PAGE Analysis – Deglycosylation Using PNGaseF

Deglycosylation was achieved using PNGase F (New England Biolabs) [14]. Aliquots of each virus concentrate (typically 1–3 µl, determined empirically) were denatured according to manufacturer’s instructions in a total reaction volume of 10 µl and samples incubated at 37°C overnight (approx. 16 hours) with 1 µl of PNGase F enzyme (neat or diluted 1/10 or 1/100) in the buffer provided by the manufacturer and 1% final concentration NP40 (provided with enzyme). Loading dye (2 µl) with 2% (v/v) β-mercaptoethanol as reducing agent was added to each sample. Samples were heated to 95°C for 3 minutes prior to loading onto NuPage™ precast 10% Bis-Tris gels and run at 125 V for 2 h using MOPS buffer (Invitrogen) followed by staining using Colloidal Blue (Invitrogen). Quantitation was carried out using a Licor Image Scanner and ImageQuant software. The content of HA for each sample was calculated as follows: firstly, the total viral protein in arbitrary units was calculated by summing the values for the HA1, HA2, NP and M bands and the HA1 and HA2 values were summed to give the HA value. The amount of HA as a percentage of total viral protein was calculated by dividing the total HA by the total viral protein, multiplied by 100.

**Figure 3:**
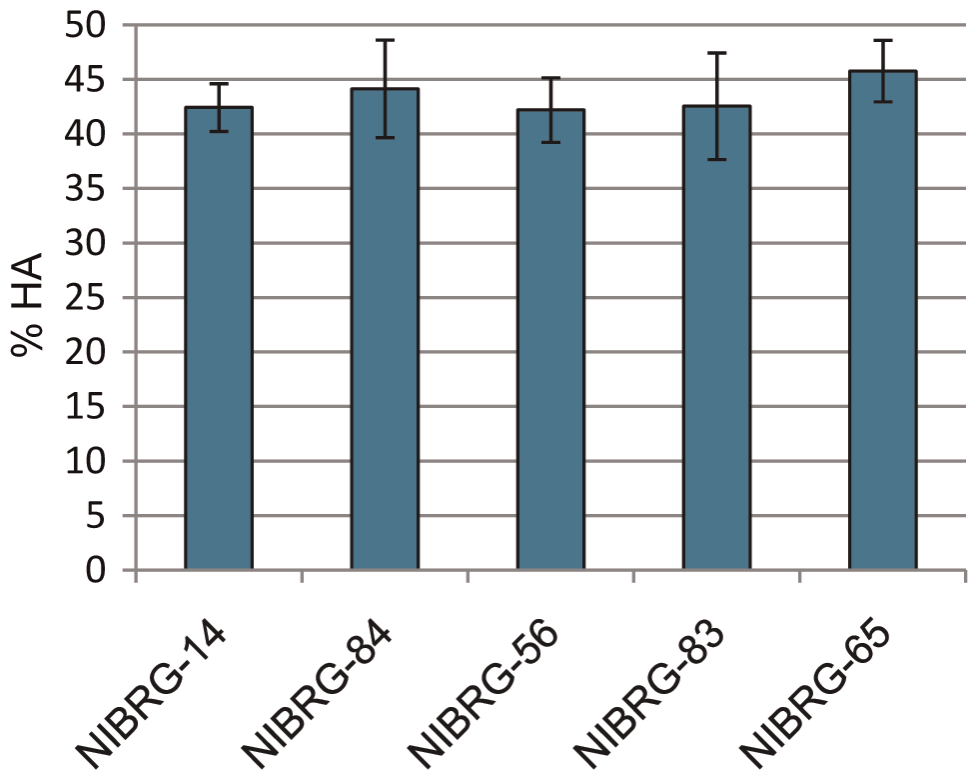
Haemagglutinin content of NIBRG-14 and panel of NIBRG-14 NCR variant viruses. Virus concentrates were analysed by SDS-PAGE following deglycosylation as described in Materials and Methods. Values are the mean of three independent virus concentrates, each analysed in triplicate. Error bars denote two standard errors from the mean and are calculated from the three means of the three independent concentrate sets for each virus.

### Growth Characteristics

Viruses at passage level VE2 (transfection in Vero cells and egg passage × 2) were diluted to 10^−4^, 10^−5^ and 10^−6^ in PBS and 100 µl aliquots of each dilution were inoculated into 10–11 day old embryonated hens’ eggs using 5 eggs per virus per dilution. Eggs were incubated at 35°C for 72 hours and virus growth in each egg assessed by haemagglutination assay following standard protocols and using 0.7% turkey erythrocytes in PBS.

For each virus, a sample of highest titre allantoic fluid from the growth experiment described above was analysed by plaque assay on MDCK cells. Samples were diluted 10^−4^, 10^−5^ and 10^−6^ in PBS and 200 µl of each dilution was used to infect wells of a six well plate, seeded with MDCK cells. Plates were incubated at room temperature for 40 minutes to allow virus absorption. Plates were overlaid with a media/Avicel suspension (1× MEM, 0.2% BA, 1× L-glutamine, 0.01% Dextran, 0.0001% TPCK Trypsin, 0.18% Sodium Bicarbonate, 1.2% Avicel type RC-581) and incubated for 48–72 hours at 35°C with 5% CO_2_ after which time the overlay was removed and cells were fixed with 3% formaldehyde solution. Cells were stained with naphthalene black.

For the growth kinetics experiment, embryonated eggs were infected with a 10^−5^ dilution of each virus stock and incubated at 35°C for 12, 24, 36, 48 and 72 hours (five eggs per time point and virus). Titres were determined using the haemagglutination assay as described above and are shown as median HA titres.

### Statistical Analysis

Statistical analysis was performed on raw data using Minitab 15 statistical software. Analysis of variance (ANOVA) was performed using a general linear model with the Tukey method for pair-wise comparisons [15].

## Results

### Comparison of NIBRG-14 HA and A/Vietnam/1194/2004 3′NCRs

For the rapid development of an H5N1 candidate vaccine virus in 2004, the modified HA and the NA genome segments of A/Vietnam/1194/2004 had to be cloned into a reverse genetics plasmid with no prior knowledge of their true NCR sequences. Thus, primers were used in PCR that had been used successfully to clone the HA genome segment of A/Hong Kong/213/2003 [10]. These primers were based on a consensus of publicly available (at that time) HA NCR sequences of highly pathogenic H5N1 viruses. The HA and NA plasmids derived from A/Vietnam/1194/2004 were then used immediately to create the CVV NIBRG-14 by reverse genetics prior to their sequence confirmation [10]. Subsequent sequence analysis showed that the HA plasmid clone used for rescuing NIBRG-14 contained a single nucleotide change at position 14 (C vs. G) of the 3′NCR compared to the synthetic primer sequence (Fig. 1). Furthermore, the sequence of the HA 3′NCR of the rescued virus, NIBRG-14, subsequently determined by the RNA ligation approach, was different again with a substitution G to A at position 14 compared to the plasmid DNA sequence from which it was derived (Fig. 1).

We then determined the true sequences of the NCRs of the HA gene of the *wt* virus A/Vietnam/1194/2004, in order to compare them to the sequences present in its derivative NIBRG-14. The sequence of the parental *wt* 3′NCR was unique and differed at two positions, 14 and 16, from that of NIBRG-14: ^14^CAAG^17^ vs. ^14^AAGG^17^, respectively (Fig. 1). The sequences of the 5′NCRs of both viruses were identical (Fig.2).

### Generation of a Panel of NIBRG-14 3′ NCR Variant viruses

Comparison of the NIBRG-14 HA 3′NCR with H5 HA 3′NCR sequences reported in publically accessible databases showed that the NIBRG-14 sequence did not conform to the majority of these. We therefore generated two variant NIBRG-14 viruses (NIBRG-56 and NIBRG-83) containing the two most frequently occurring 3′NCR sequences (^14^CAGG^17^ and ^14^CAUA^17^, respectively) of highly pathogenic H5N1 viruses. We also generated a third variant with an HA 3′NCR which was identical to that of *wt* A/Vietnam/1194/2004 (NIBRG-84; ^14^CAAG^17^). The sequences of the 3′NCRs of the HA RNA segments of these four NIBRG-14 derived viruses were determined by the RNA ligation approach and found to be equivalent to the primers used during their generation (Figs 1 and 2).

**Figure 4:**
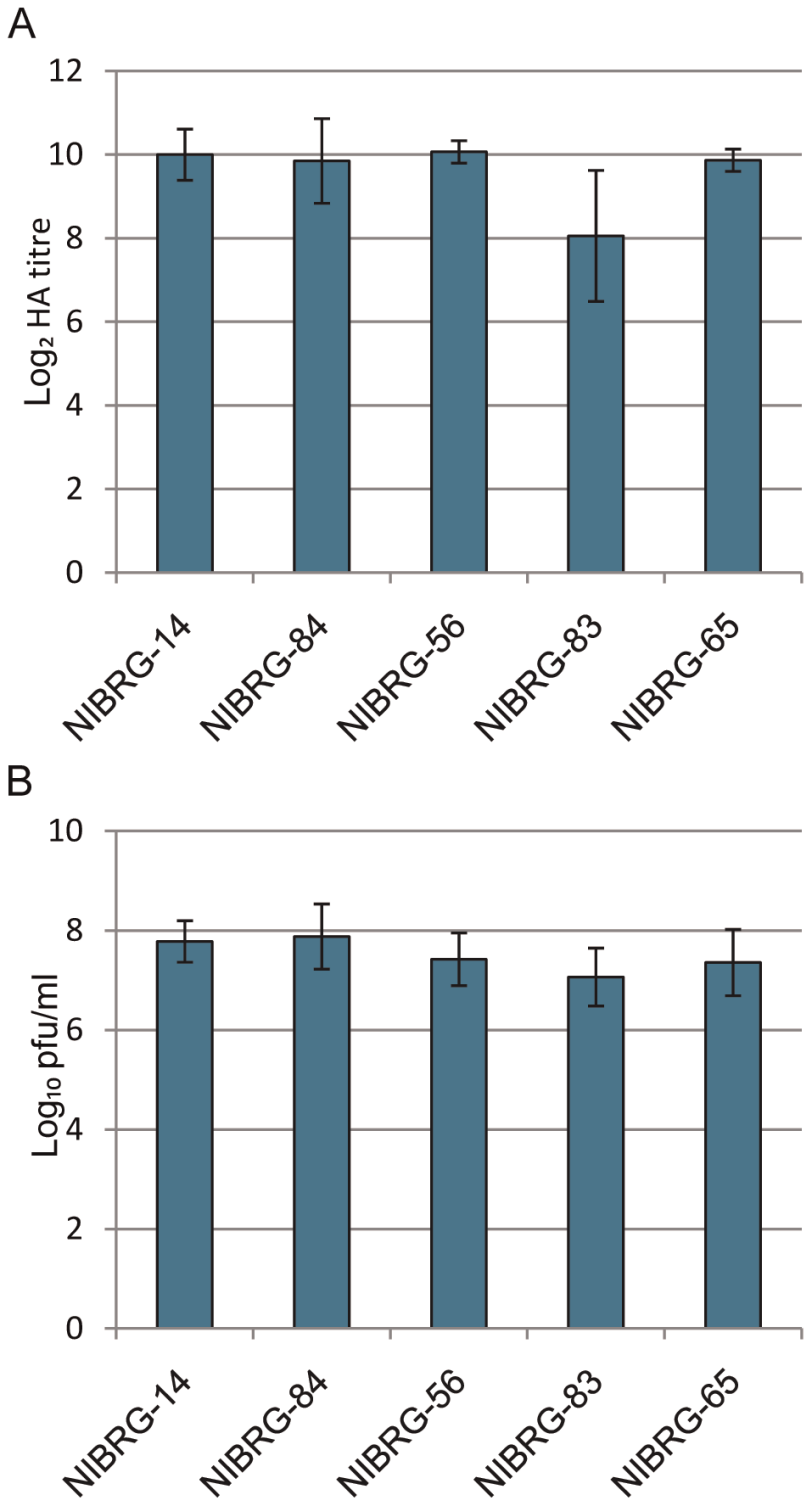
Final titres of NIBRG-14 and the panel of NIBRG-14 NCR variant viruses. (A) Embryonated hens’ eggs were infected with dilutions (10^−4^, 10^−5^, 10^−6^) of each virus (five eggs per dilution, three independent experiments). Values are the log_2_ means of the 10^−5^ dilution haemagglutination titres based upon three independent experiments. Error bars denote two standard errors from the mean and are calculated from the three experimental means for each virus. (B) Allantoic fluids from the dilution giving highest mean HA titres for each virus from the experiment shown in (A) were used to determine infectivity titres, as described in Materials and Methods. Values are the means of the log_10_ pfu/ml for each virus based on the means of the 3 independent experiments. Error bars represent 2 standard errors from the mean and are calculated from the three experimental means for each virus.

### HA Content of NIBRG-14 NCR Variant Viruses

It was reported previously by us and others that yields of viral protein and HA antigen of NIBRG-14 were low compared to other candidate vaccine viruses [14], [16]. It was thus of interest to investigate whether or not the sequence of the HA 3′NCR influences yield and growth properties of NIBRG-14. In addition to the viruses described above containing up to three nucleotide differences in the 3′ NCR as compared to NIBRG-14 (NIBRG-56, NIBRG-83, NIBRG-84), virus NIBRG-65 which contains the HA open reading frame of NIBRG-14 flanked by the 3′ and 5′ NCRs of the HA of PR8 as described previously, was included in this analysis [12]. Virus concentrates of all viruses were prepared and the relative HA content, i.e. the amount of HA as a proportion of total viral protein, was assessed using SDS-PAGE analysis of deglycosylated samples [14]. No significant differences between the viruses were found in this analysis (Fig. 3).

### Growth Characteristics of NIBRG-14 NCR Variant Viruses

A range of virus dilutions was used to inoculate embryonated hens’ eggs and growth was assessed by haemagglutination assay (Fig 4A). Using a general linear model (ANOVA), NIBRG-83 was found to have a statistically lower haemagglutination assay titre than NIBRG-14, NIBRG-56, NIBRG-65 and NIBRG-84 (P<0.0001). No other pair-wise comparisons were statistically different.

The allantoic fluids harvested from this growth study were used to determine infectivity titres (in pfu/ml) by plaque assay on MDCK cells (Figure 4B). Using a general linear model (ANOVA), statistical differences were found between some of the viruses. Both NIBRG-14 and NIBRG-84 were found to have significantly higher infectivity titres than NIBRG-56 (p = 0.0259 and 0.0021, respectively), NIBRG-65 (p = 0.003 and 0.0002, respectively) and NIBRG-83 (p<0.0001 when compared to both NIBRG-14 and NIBRG-84), whilst NIBRG-56 was significantly higher than NIBRG-83 (p = 0.0369). Although these significant differences were found, the level of variability observed between the viruses was similar to the variability observed between experiments (estimated standard deviations of 0.31 and 0.46 of a log respectively). Furthermore, the estimated standard deviation between replicates was 0.32 of a log which is on a similar level to the variability between viruses. The largest difference in means between viruses was between NIBRG-83 and NIBRG-84, with a difference of 0.81 of a log and a 95% confidence interval of 0.47 to 1.16.

**Figure 5:**
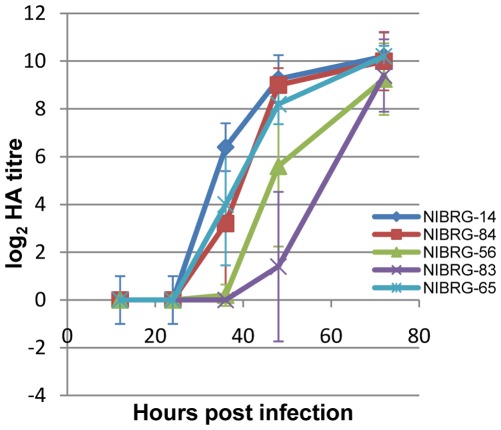
Growth kinetics of NIBRG-14 and the panel of NIBRG-14 NCR variant viruses. Embryonated hens’ eggs were infected with a 10^−5^ dilution of each virus and incubated for various times. Titres at each time point were determined using the haemagglutination assay as described in Materials and Methods. Values are the log_2_ median HA titres; error bars denote standard deviations.

To determine the kinetics of growth of these viruses, embryonated eggs were infected and haemagglutination titres of allantoic fluids were determined at 12, 24, 36, 48 and 72 hours post infection (Fig. 5). The kinetics of virus growth differed between the viruses with statistically significant differences observed at 36 h.p.i. (NIBRG-56 and NIBRG-83 significantly different from NIBRG-14) and 48 h.p.i. (NIBRG-83 significantly different from NIBRG-14 and NIBRG-83); however, at 72 h.p.i. all viruses had attained a similar final HA titre.

Overall, while the changes made to the 3′ NCR of NIBRG-14 in some cases led to statistically significant differences, we concluded that these differences were not sufficient biologically to warrant further investigation. Interestingly, none of the 3′ NCR variant viruses generated in this study improved the growth of NIBRG-14.

## Discussion

Sequencing of the coding regions of influenza A virus genome segments is technically straightforward whilst extra efforts are required to deduce the sequence of the terminal NCRs. Consequently, researchers who focus on the coding regions of the influenza virus genome tend to ignore the NCRs despite them containing important cis-acting signals that affect various stages of the viral life cycle, such as replication, transcription and genome packaging. Even when sequence information is available in the public domain for the NCRs, it is often unclear whether or not these terminal sequences are ‘genuine’, i.e. whether they are derived from sequencing the viral RNA (or cDNA copies thereof), or whether they are primer-derived artefacts that do not necessarily reflect the sequence present in the virus itself.

The lack of available NCR sequences and the lack of information regarding the derivation of NCR sequences when they are presented, constitute a problem for the design of primers used for the segment-specific amplification of novel influenza viruses. Therefore, primers may be designed based on information that is incorrect or inaccurate. In the case of the H5N1 CVV NIBRG-14, no information about the sequence of the NCRs was available at the time the virus was generated through reverse genetics technology.

While NIBRG-14 has been widely used, including for the production of vaccine lots for clinical trials [17], [18], it has long been recognised that yields obtained from this virus are less than optimal [14], [16]. In this study, we investigated whether the use of altered NCRs, particularly those reflective of the *wt* parental virus or of other wt H5N1 viruses, improved growth and yield properties of the CVV.

No significant differences were found between the viruses that had specific defined variations in their 3′NCR in terms of HA content, a relevant marker for vaccine yield [12], [19], and none of the NIBRG-14 NCR variant viruses showed improved growth compared to NIBRG-14. Conversely, while some viruses showed somewhat reduced final infectivity titres and slightly delayed growth kinetics, changes to the 3′ NCR did not massively reduce the growth potential of NIBRG-14 in embryonated chicken eggs. These findings may serve to assure the influenza vaccine community that generation of CVVs using best-guess NCR sequences, based on sequence alignments, are likely to produce robust viruses that need not be changed if it is subsequently found that their NCR sequences differ from the respective wt parental virus(es). However, it would be useful if influenza sequences deposited in publically accessible databases contained clear annotation as to which portions of sequence are truly virus-derived and which are primer-derived, if any.

In conclusion, we have found that minor alterations of the 3′NCR of an H5N1 CVV had minimal impact on its growth and HA yield characteristics. Further studies will be required to establish whether more dramatic sequence changes involving both non-coding and coding regions, similar to those described recently [12], [19], may be beneficial for vaccine production of a wide variety of haemagglutinin subtypes.
