# Correction: Permissible Variation in the 3′ Non-Coding Region of the Haemagglutinin Genome Segment of the H5N1 Candidate Influenza Vaccine Cirus NIBRG-14

**DOI:** 10.1371/annotation/1fcf03a1-a452-414f-bf45-919a059a7ba7

**Published:** 2012-06-05

**Authors:** Rachel E. Johnson, Michelle Hamill, Ruth Harvey, Carolyn Nicolson, James S. Robertson, Othmar G. Engelhardt

There was a spelling error in the title. The correct title is: Permissible Variation in the 3′ Non-Coding Region of the Haemagglutinin Genome Segment of the H5N1 Candidate Influenza Vaccine Virus NIBRG-14. The correct citation is: Johnson RE, Hamill M, Harvey R, Nicolson C, Robertson JS, et al. (2012) Permissible Variation in the 3′ Non-Coding Region of the Haemagglutinin Genome Segment of the H5N1 Candidate Influenza Vaccine Virus NIBRG-14. PLoS ONE 7(5): e36241. doi:10.1371/journal.pone.0036241 

